# 515 Retrospective Review of Silver Sulfadiazine use in Sulfa Allergic Patients at a Regional Burn Center

**DOI:** 10.1093/jbcr/irad045.112

**Published:** 2023-05-15

**Authors:** Kristina Gemayel, Mariel McLaughlin, Jake Laun, David Smith

**Affiliations:** Department of Plastic Surgery, University of South Florida, Tampa, Florida; Department of Plastic Surgery, University of South Florida, Tampa, Florida; Department of Plastic Surgery, University of South Florida, Tampa, Florida; Department of Plastic Surgery, University of South Florida, Tampa, Florida; Department of Plastic Surgery, University of South Florida, Tampa, Florida; Department of Plastic Surgery, University of South Florida, Tampa, Florida; Department of Plastic Surgery, University of South Florida, Tampa, Florida; Department of Plastic Surgery, University of South Florida, Tampa, Florida; Department of Plastic Surgery, University of South Florida, Tampa, Florida; Department of Plastic Surgery, University of South Florida, Tampa, Florida; Department of Plastic Surgery, University of South Florida, Tampa, Florida; Department of Plastic Surgery, University of South Florida, Tampa, Florida; Department of Plastic Surgery, University of South Florida, Tampa, Florida; Department of Plastic Surgery, University of South Florida, Tampa, Florida; Department of Plastic Surgery, University of South Florida, Tampa, Florida; Department of Plastic Surgery, University of South Florida, Tampa, Florida

## Abstract

**Introduction:**

Plastic Surgeons are commonly called upon to evaluate wounds. Most wounds in otherwise healthy individuals will heal with minimal intervention, however in patients with comorbid conditions and systemic disease the incidence of nonhealing wounds is much more prevalent. Silver sulfadiazine is the most common topical antimicrobial used in burn management. Current literature recommends avoiding the use of silver sulfadiazine in burn patients with sulfa allergies. The intent of this retrospective review is to examine the safety of silver sulfadiazine use in documented sulfa allergic burn patients at a level one trauma hospital and regional burn center.

**Methods:**

After obtaining IRB approval for electronic chart review of patients who were treated with silver sulfadiazine, charts were reviewed and exclusion criteria applied. Patients included in the study had documented burns treated by burn team providers, application of topical silver sulfadiazine for primary burn management, and a documented sulfa allergy. Chart review identified 70 patients who met the stated criteria out of 2,644 patients treated over a five year period.

**Results:**

None of the 70 patients with documented sulfa allergies suffered adverse reactions to silver sulfadiazine use for primary burn care. There were no documented systemic or anaphylactic reactions, hives, or additional adverse reactions after administration. No cessation of the medication was documented due to intolerance.

**Conclusions:**

Silver sulfadiazine is a common topical antimicrobial treatment in acute burn care. It has previously been avoided in patients with documented sulfa allergy due to concern for cross reactivity. However, in this population of patients, there were no documented adverse reactions to topical silver sulfadiazine. This study suggests that withholding topical sulfonamides from patients with prior reactions to antibacterial sulfonamides may not be clinically justified.

**Applicability of Research to Practice:**

We present evidence in our retrospective review that administration of topical silver sulfadiazine for primary burn care can be done in a safe manner, without adverse allergic reactions.

